# Supplementary material for: Static and Resonant Properties and Magnetic Phase Diagram of LiMn2TeO6
Source: Materials (Basel). 2022 Dec 6;15(23):8694. doi: 10.3390/ma15238694 (PMC9735636; doi:10.3390/ma15238694)
Supplement: Supplementary file 1 [file materials-15-08694-s001.zip › materials-2014585-supplementary.pdf]

Supplementary Materials

for the article

Complex spin ordering and magnetic phase diagram of $LiMn_2TeO_6$

Tatyana Vasilchikova,[1,5] Evgeniya Vavilova,[2] Timur Salikhov,[2] Vladimir Nalbandyan,[3] Shanu Dengre,[4] Rajib Sarkar,[4] Hans-Henning Klauss,[4] Alexander Vasiliev[1,5]

[1]*Lomonosov Moscow State University, 119991 Moscow, Russia*
[2]*Zavoisky Physical-Technical Institute, FRC Kazan Scientific Center of RAS, 420029 Kazan, Russia*
[3]*Southern Federal University, 344090 Rostov-on-Don, Russia*
[4]*Institute for Solid State and Material Physics, Technische Universität Dresden, 01069 Dresden, Germany*
[5]*National University of Science and Technology "MISiS", 119949 Moscow, Russia*

*Corresponding author: anvas2000@yahoo.com

S1. *ac* susceptibility

The frequency shift of the T_f, i.e. the peak shift per decade of frequency ($\omega = 2\pi f$) can by quantified by the Mydosh parameter [S1]:

$$K = \frac{\Delta T_f}{T_f \Delta(\log \omega)}$$

This parameter allows to distinguish a spin-glass behavior from a superparamagnet one. The K value has been categorized as 0.005 – 0.01 for spin glass, ~ 0.03 − 0.06 for cluster glass, and > 0.1 for superparamagnetic compounds [S1]. For $LiMn_2TeO_6$ we estimated K as 0.03, which falls in the range expected for cluster glasses.

The variation of the freezing temperature T_f with the relaxation time τ can be analyzed with a critical slowing down model [S2-S4]. The frequency dependence of T_f data fits well with the power law [S2-S4]:

$$\tau(T_f) = \tau_0 \left(\frac{T_f}{T_g} - 1 \right)^{z\nu}$$

as shown on the Figure S1, where τ_0 is microscopic spin flipping time, T_f is frequency dependent freezing temperature at which the maximum relaxation time ($\tau = 1/\omega$) of the system correspond to the measured frequency, T_g is glassy freezing temperature (as $f \rightarrow 0$) and $z\nu$ denote the critical exponent. Typically, the value τ_0 lies within the range of 10^{-10} to 10^{-14} s, and the exponent $z\nu$ ranging from 4 to 12 [S1-S5]. The best fit of our experimental data yielded the following quantities: T_g = 14 K, $\tau_0 = 10^{-11}$ s, and $z\nu$ = 7. Obviously, these values are in good agreement with those for well-known manganese spin cluster and reentrants spin glass materials [S4, S6-S11].

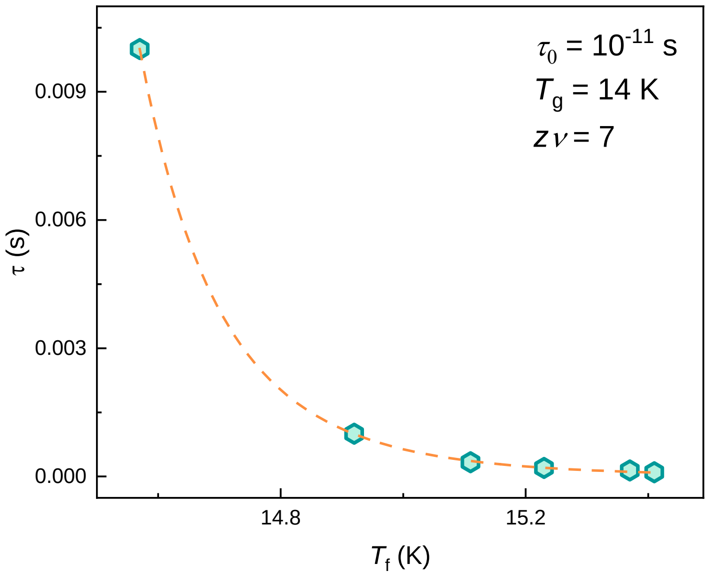

Figure S1. Critical slowing down behavior of the peak position T_f.

S2. NMR

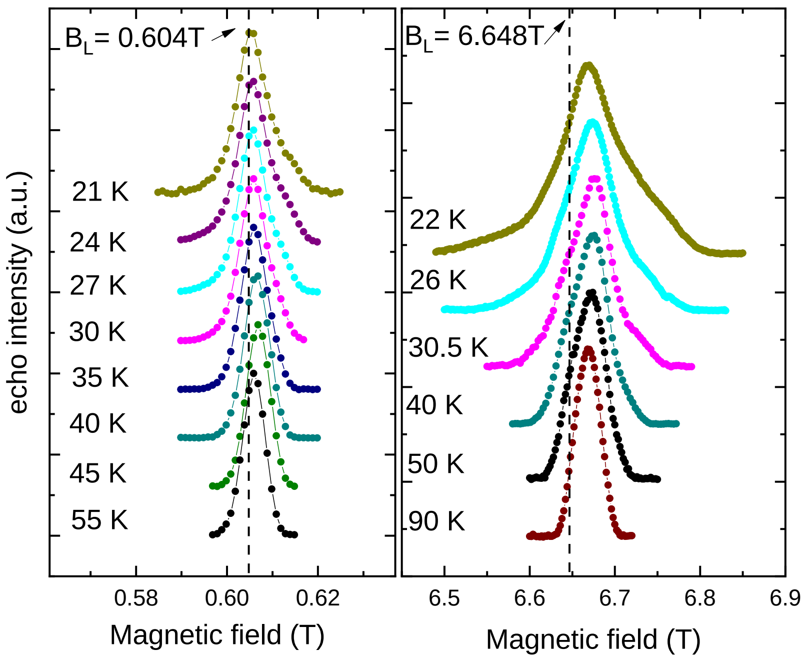

Figure S2. ^{7}Li NMR spectrum for $LiMn_2TeO_6$ at different temperatures in low and high external fields.

The observed ^{7}Li NMR spectrum (see Fig. S2) is a superposition of the signals from all lithium nuclei in the powder sample. The structure of the ^{7}Li NMR line in the paramagnetic temperature

region is smeared out by the random distribution of the crystallographic and the hyperfine field tensor axes in the powder sample and spectra have atypical slightly asymmetric powder lineshape. ^7Li has a nuclei spin $I = 3/2$ and a quadrupole moment -4.01×10^{-30} m^2. However, the quadrupole splitting is not resolved in the inhomogeneously broadened spectrum of the powder sample. The line shift k was determined from the position of the maxima of the spectrum H_{max} as $k = 100\%$ $(H_{max} - H_L)/H_L$, where $H_L = \omega_L/\gamma^7_{Li}$ and ω_L is a Larmor frequency.

References

S1. Mydosh, J. A. *Spin Glasses, An Experimental Introduction*; Taylor & Francis: London, 1993.
S2. Mydosh, J. A. Spin glasses: redux: an updated experimental/materials survey. *Rep. Prog. Phys.* **2015**, *78, 052501*. https://doi.org/10.1088/0034-4885/78/5/052501.
S3. Binder, K.; Young, A. P. Spin glasses: Experimental facts, theoretical concepts, and open questions. *Rev. Mod. Phys.* **1986**, *58, 801-976*. https://doi.org/10.1103/RevModPhys.58.801.
S4. Chun, S. H.; Lyanda-Geller, Y.; Salamon, M. B.; Suryanarayanan, R.; Dhalenne, G.; Revcolevschi, A. Reentrant spin glass behavior in layered manganite $La_{1.2}Sr_{1.8}Mn_2O_7$ single crystals, *Journal of Applied Physics* **2001**, *90, 6307-6311*. https://doi.org/10.1063/1.1419260.
S5. Balanda, M. AC Susceptibility Studies of Phase Transitions and Magnetic Relaxation: Conventional, Molecular and Low-Dimensional Magnets. *Acta Phys. Pol.* **2013**, *A 124, 964-967*. https://doi.org/10.12693/APhysPolA.124.964.
S6. Gunnarsson, K.; Svedlindh, P.; Nordblad, P.; Lundgren, L.; Aruga, H.; Ito, A. Dynamics of an Ising Spin-Glass in the Vicinity of the Spin-Glass Temperature. *Phys. Rev. Lett.* **1988**, *61, 754-757*. https://doi.org/10.1103/PhysRevLett.61.754.
S7. Kumar, N. Rajeesh; Karthik, R.; Vasylechko, L.; Selvan, R. Kalai. Reentrant spin-glass behaviour in highly frustrated Mn-rich spinel zinc manganate. *J. Phys.: Condens. Matter* **2020**, *32, 245802*. https://doi.org/10.1088/1361-648X/ab786e
S8. Ghosh, D.; Duttaa, U.; Haque, A.; Mordvinova, N.; Lebedev, O.; Pal, K.; Gayen, A.; Mahata, P.; Kundu, A.; Seikh, M. Evidence of low temperature spin glass transition in bixbyite type $FeMnO_3$. *Materials Science & Engineering B* **2017**, *226, 206-210*. https://doi.org/10.1016/j.mseb.2017.09.022.
S9. Kolesnik, S.; Dabrowski, B.; Chmaissem, O. Structural and physical properties of $SrMn_{1-x}Ru_xO_3$ perovskites. *Phys. Rev. B* **2008**, *78, 214425*. https://doi.org/10.1103/PhysRevB.78.214425.
S10. Jeon, B.-G.; Yoo, K.; Kim, K.; Sundaresan, A. Reentrant spin-glass state and magnetodielectric effect in the spiral magnet $BiMnFe_2O_6$. *Phys. Rev. B* **2014**, *90, 024413*. https://doi.org/10.1103/PhysRevB.90.024413.
S11. Greedan, J. E.; Raju, N. P.; Maignan, A.; Simon, Ch.; Pedersen J. S.; Niraimathi, A. M.; Gmelin, E.; Subramanian, M. A. Frustrated pyrochlore oxides, $Y_2Mn_2O_7$, $Ho_2Mn_2O_7$, and $Yb_2Mn_2O_7$: Bulk magnetism and magnetic microstructure. *Phys. Rev. B* **1996**, *54, 7189-7200*. https://doi.org/10.1103/PhysRevB.54.7189.
